# Use of Sensor Array Analysis to Detect Ovarian Cancer through Breath, Urine, and Blood: A Case-Control Study

**DOI:** 10.3390/diagnostics14050561

**Published:** 2024-03-06

**Authors:** Roberto Angioli, Marco Santonico, Giorgio Pennazza, Roberto Montera, Daniela Luvero, Alessandra Gatti, Alessandro Zompanti, Panaiotis Finamore, Raffaele Antonelli Incalzi

**Affiliations:** 1 Unit of Gynecology, University Campus Bio-Medico of Rome, Via Alvaro del Portillo 200, 00128 Rome, Italy; r.angioli@policlinicocampus.it (R.A.); a.gatti@unicampus.it (A.G.); 2 Unit of Electronics for Sensor Systems, Department of Science and Technology for Sustainable Development and One Health, University Campus Bio-Medico of Rome, Via Alvaro del Portillo 21, 00128 Rome, Italy; 3 Unit of Electronics for Sensor Systems, Department of Engineering, University Campus Bio-Medico of Rome, Via Alvaro del Portillo 21, 00128 Rome, Italy; 4 Unit of Geriatrics, University Campus Bio-Medico of Rome, Via Alvaro del Portillo 200, 00128 Rome, Italy

**Keywords:** ovarian cancer, sensor array, carbohydrate antigen 125, human epididymis protein 4

## Abstract

Ovarian cancer (OC) is the eighth most common cancer in women. Since screening programs do not exist, it is often diagnosed in advanced stages. Today, the detection of OC is based on clinical examination, transvaginal ultrasound (US), and serum biomarker (Carbohydrate Antigen 125 (CA 125) and Human Epididymis Protein 4 (HE4)) dosage, with a sensitivity of 88% and 95%, respectively, and a specificity of 84% for US and 76% for biomarkers. These methods are clearly not enough, and OC in its early stages is often missed. Many scientists have recently focused their attention on volatile organic compounds (VOCs). These are gaseous molecules, found in the breath, that could provide interesting information on several diseases, including solid tumors. To detect VOCs, an electronic nose was invented by a group of researchers. A similar device, the e-tongue, was later created to detect specific molecules in liquids. For the first time in the literature, we investigated the potential use of the electronic nose and the electronic tongue to detect ovarian cancer not just from breath but also from urine, blood, and plasma samples.

## 1. Introduction

According to the Global Cancer Observatory, ovarian cancer is the eighth most common cancer in women, with 313.959 new cases and 207.252 new deaths worldwide in 2020 [1]. In Italy, 5370 new cases and 3285 deaths were registered last year [1]. Most cases, around 75% [2], are diagnosed in advanced stages, thus making ovarian cancer a particularly lethal disease, named the “big silent killer”. This happens because of its rapid spread through the peritoneal surface, but also because of the lack of valid screening programs, in contrast to other gynecological diseases. For example, cervical cancer has finally become a preventable cancer nowadays, thanks to the Papanicolaou screening test, the Human Papilloma Virus (HPV) vaccination, and other effective interventions [3]. Ultrasound and hysteroscopy are of paramount importance for the prevention and early detection of endometrial cancer, along with serum biomarkers CA125 and HE4, which could help in the diagnosis. Conversely, no method is really effective in detecting ovarian cancer at early stages, and its prognosis remains poor. Not even its signs and symptoms are reliable since they occur when the disease has already spread. In clinical practice, clinical examination, transvaginal ultrasound (US), and serum biomarkers, such as Carbohydrate Antigen 125 (CA125) and Human Epididymis Protein 4 (HE4) dosage, are used to investigate the presence of ovarian cancer (OC), with a sensitivity of 88% and 95% and a specificity of 84% and 76% respectively, but these data are not enough, and several early stages are missed [4,5,6,7]. The aim of transvaginal ultrasound is to observe both ovaries, calculate their volume, and report the presence of abnormal lesions. Any variation in morphology or increase in volume or vascularization needs to be further explored [7]. Unfortunately, ultrasounds are extremely operator-dependent, and while CA125 may be elevated in OC, it may also be elevated in endometriosis or other benign diseases and thus has a low specificity [5]. HE4 reaches a specificity of 86% [5,8,9], and it is usually dosed with CA125. HE4 is approved by the Food and Drug Administration in the United States as a reliable tumor biomarker for ovarian cancer. Nonetheless, HE4 can also be elevated in renal diseases, and this again reduces its specificity. Therefore, it is clear that all these methods have several limitations; as a consequence, the great challenge now is to find an effective screening test for OC and develop a strong prevention. Many scientists have recently focused their attention on volatile organic compounds (VOCs). These are gaseous molecules easily collected from the breath because they pass from the bloodstream into the lungs and also from the blood into the urine. They might provide interesting information on several diseases, such as renal dysfunctions, asthma, and solid tumors [10,11,12,13,14,15]. The original idea was born in 2008: several studies by Horvath et al. demonstrated that human ovarian cancer has a specific odor that can be detected by a trained dog. In addition, the same dog was able to distinguish among several histopathological types and grades, as well as healthy control samples, with a sensitivity of 100% both in tissue and in blood tests and a specificity of 95% and 98% in tissue and blood tests, respectively. Unfortunately, dogs are not suitable for clinical practice [16,17,18]. Based on their discovery, Horvath et al. used an electronic nose that can accomplish the same task, with a sensitivity of 84.4% and a specificity of 86.8% in tissue tests [16,17,18]. This electronic nose was a combination of four gas sensors, operating at different temperatures, made of metal oxide to better differentiate between various gases. The nose produces some signals, which are reduced to numbers and analyzed through the Weka algorithm package [16,17,18]. At our university, Campus Bio-Medico of Rome, a new ongoing study is using the sensor array analysis (e-nose) to detect lung cancers, while a similar study on prostate cancer has already been published [19]. The potential use of the e-nose has been tested in several other solid tumors, for example, in colorectal cancer [12,13,20,21], lung cancer [11,22], and breast cancer [23]. Regarding ovarian cancer, there are at least a couple of articles to be mentioned. Haick et al., in 2014 [24,25], published a pilot study that aimed to detect ovarian cancer through exhaled breath samples. The nanoarray measured volatile organic compounds with a good sensitivity (79%) and a 100% specificity in detecting patients with ovarian cancer vs. controls. It also discriminated between early and advanced ovarian cancers with good accuracy. Inspired by these previous studies [16,17,18,26,27], Raspagliesi et al. [26] published in 2020 a prospective study demonstrating the ability of the e-nose to discriminate between breath samples from patients with ovarian cancer and controls. In addition, it identified four sensors involved in ovarian cancer detection that were also able to discriminate between early-stage and advanced-stage ovarian cancer [26]. Along with the e-nose, another important discovery was the electronic tongue. The operating mechanism is similar. The e-tongue is a sensor array that produces an electrical response according to the material tasted, which serves as a fingerprint for the analyzed sample. This tool was used for various applications, first to classify food, wine, and milk or to detect impurities or pollutants in water [27]. Then, its use was extended to detect other diseases including tumors, and it was tested on urine samples from male patients with bladder cancer [28]. This study, by Lvova et al., suggested that the e-tongue could discriminate tumor samples from controls. However, the numbers were too low though (17 cancers and 10 controls), so further research was needed [28]. In 2016, Pascual et al. introduced the usage of the e-tongue to detect patients with prostate cancer. This tool recognized an electrochemical fingerprint in urine samples with a sensitivity of 91% and a specificity of 73% [29]. In particular, the e-tongue correctly classified 20 out of 22 samples of patients with prostate cancer and 11 out of 15 samples of controls. [29] This specificity and sensibility were even higher than the prostate-specific antigen (PSA) test in blood, which was the main procedure to detect prostate cancer up to now. The PSA sensitivity and specificity are 34.9% and 63.1% respectively, when using a 4.0 ng/mL cutoff value [30]. The e-tongue used by Pascual was an array of seven metal wire electrodes housed inside a steel cylinder. A Large Amplitude Pulse Voltammetry (LAPV) waveform was applied to each electrode, and the resulting currents versus time profile for each electrode was measured [29]. Therefore, the e-tongue has been tested on urine samples from bladder and prostate cancer, as already mentioned, but it has never been tested on urine samples from ovarian cancer patients up to now. For the very first time in literature, we tested the e-tongue in this field. In our study, the electronic tongue was used not only on urine samples but also on blood and plasma samples from ovarian cancer women. In addition, we collected breath samples from the same patients to be analyzed by the electronic nose. To sum up, we merged the e-tongue data (made of 1500 sensors divided into three blocks of 500) with the e-nose data (made of 32 sensors) to increase the sensitivity and specificity for better detection of ovarian cancer. As a secondary endpoint, we correlated the fingerprints obtained from the e-nose and e-tongue with Carbohydrate Antigen 125 (CA125) and Human Epididymis Protein 4 (HE4) serum biomarkers collected before the surgical operation. This is also a first in the field of sensor arrays. 

## 2. Materials and Methods

**Figure 1:**
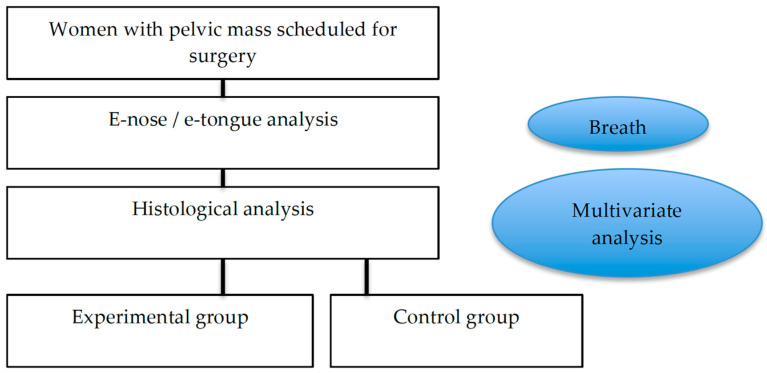
Flowchart of this study.

We enrolled patients between 2017 and 2019 who were affected by an ovarian mass, suitable for pelvic surgery, and referred to the University Campus Bio-Medico of Rome. The women underwent surgery according to the inclusion and exclusion criteria, in a double-blind analysis (see the flowchart in Figure 1). In the first part of this study (training analyses), the population was divided into two main groups according to the pathology report obtained by surgery: (1) control: women with adnexal masses with evidence of benign disease at histological evaluation (2) OC group: women with malignant adnexal masses.

The inclusion criteria were: one or multiple ovarian masses, age between 18 and 80 years, a good performance status (ECOG < 2), and written informed consent. The exclusion criteria were concomitant neoplasia, non-ovarian disease at surgery, the presence of uncontrolled systemic disease, and previous surgery/chemotherapy. Breath, blood, and urine were the samples used for analysis. As soon as the patients were admitted to the hospital, we collected breath by a pneumo-pipe. Nurses collected urine and venous blood samples. We immediately spun the blood to obtain plasma (2500 rpm at 4 °C for 10 min). In the following 1 h, we analyzed all the samples including urine, plasma, blood, and VOCs. Different kinds of sensors were used as follows: liquids were analyzed through electrochemical sensors, while VOCs were analyzed through gas sensors based on quartz microbalance. Gas sensors are not selective. The transducer is a crystal quartz that can modify its oscillation frequency, adsorbing VOCs on its surface, covered with different sensing materials (anthocyanins). Liquid sensors were used to analyze chemical compounds in the solutions. The transducer is based on an electronic architecture oriented to a three-electrode system. The interactions between the electrodes and the solutions produce oxidation/reaction processes with a change in electrons. By applying different voltages on the electrodes, we obtained different current values. A total of 500 different voltage signals are applied to the electrodes, resulting in 500 current values that form a typical fingerprint for the solution. Below, a broader description of the system is provided.

### 2.1. Breath Analysis

#### 2.1.1. Gas Sensor Array

In this paper, the last version of a gas sensor array developed at the University Campus Bio-Medico di Roma was used. The device is composed of 7 quartz microbalances covered with different biological materials. Each material can detect different volatile compounds, and each compound can be detected by more sensors. This is a peculiarity for this kind of sensor in order to counteract and exploit non-selectivity. The interaction with a gas or vapor provides a characteristic pattern for the specific target gas. The transducer, as just mentioned before, is based on a mass sensor that changes its resonant frequency adsorbing a specific compound on its surface. This sensor can oscillate at a frequency of 20 MHz. The transducer drives a specific oscillator circuit able to provide a frequency response. Thanks to a dedicated electronic low-noise interface, it is possible to consider minimum detectable frequency shifting around 1 Hz. This low value is very important in obtaining a high-power resolution. The overall device can store the information obtained by the 7 sensors, and in this way, it is possible to build a specific database.

#### 2.1.2. Breath Collection System: Pneumopipe

**Figure 2:**
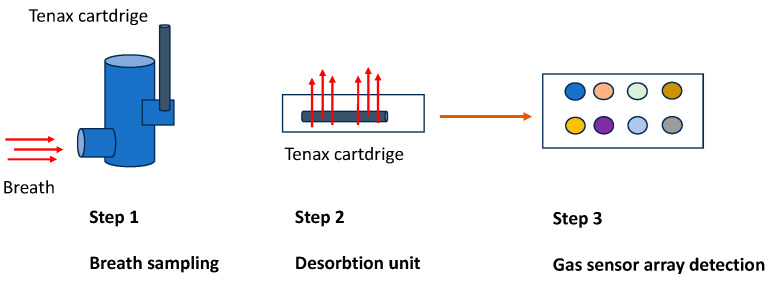
Gas collection and transportation: the flux in the cartridge is 40 mL/min, so, for 3 min, it corresponds to a volume of 120 mL/min. Regarding the details of all expired air, only a small volume is used for absorption in the cartridge (Step 1). The tenax tube is heated to different temperatures (50, 100, 150, and 200 °C) through a desorption unit (Step 2). The desorbed samples are conveyed into a chamber containing eight different types of crystal quartz that can detect the gas (Step 3).

For breath collection, a UCBM-patented system was used (European patent EP2641537 2013). The system is composed of a pneumatic device designed to reduce the resistance at expiration. The device body has size and volume dedicated to this specific application. The patient expires into a sterilized mouthpiece inside a chamber continuously for three minutes. An electronic system placed at the end of the chamber catches the volatile organic compounds, delivering the collected mixture in a tenax cartridge. The cartridge contains a sensing material able to adsorb the volatile organic compounds. The cartridge can be transported, stored, and measured off-line. For this specific application, the cartridge was placed in a desorption unit (DU) connected to the gas sensor array described before. The desorption of the sensing material is obtained through a temperature profile using nitrogen as a carrier gas. Different temperatures have the role of potentiating and differentiating the sensitivity of sensors. Using four temperatures, it is possible to obtain an array of 32 virtual sensors for each breath specimen (Figure 2).

#### 2.1.3. Liquid Sensor Array

Biological fluids (blood, plasma, and urine) were measured by a liquid sensor array, the e-tongue. This kind of sensor is used to analyze chemical compounds in solutions. The transducer is based on an electronic architecture oriented to the following three-electrode system: reference (Ag), counter (Pt), and working (Au). The interactions between the electrodes and the solutions produce oxidation/reaction processes with a change in electrons. By applying different voltages on the electrodes, we obtained different current values. A total of 500 different voltage signals were applied to the electrodes, resulting in 500 current values that form a typical fingerprint for the solution. The electrodes are not functionalized, and it is possible to obtain different fingerprints for different solutions. The current is converted into a voltage through a trans-impedance electronic circuit. The reproducibility is guaranteed by a dedicated electronic interface developed by a unit of electronics for sensor systems of Università Campus Bio-Medico di Roma. A multivariate data analysis must be used to classify the samples.

#### 2.1.4. Data Analysis

A PLS-DA model was obtained using a data fusion among different kinds of sensors as follows: 

Five hundred values related to the electrochemical sensors for blood.

Five hundred values related to the electrochemical sensors for urine.

Five hundred values related to the electrochemical sensor for plasma.

Thirty-two values related to the responses of gas sensors (QMB).

The classification models were obtained considering the overall data for each biological fluid. The data were trained on clinical information provided by medical equipment.

**Figure 3:**
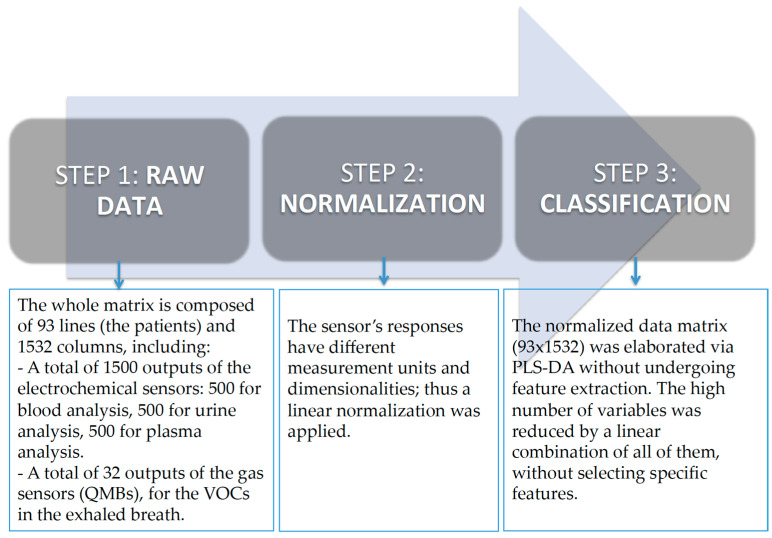
Data analysis.

The single models obtained considering different biological fluids were not significant in terms of percentage of correct classification. It is important to remember that the method’s power considers all the data. This is because of the fact that each sensor is not selective to specific compounds (solution or VOCs). Thus, each biological fluid is not able to provide specific information for the pathology if analyzed with a single kind of sensor. Multivariate data analysis was applied to the multi-sensor results to obtain a typical model that had the features extracted by the several approaches described above as input and specific information about the process under investigation as output. The signal that resulted from the VOC and liquid measurements was processed into a matrix of features that is suitable for statistical analyses. Exploratory (supervised) data analysis and partial least square discriminant analysis (PLS-DA) were used. This kind of analysis provides a predictive model between the sensor responses and the clinical data (Figure 3). The leave-one-out criterion was performed for the validation test. The software used for statistical analysis was PLS Toolboox Version 7.3 (2013) (Eigenvector Research, Inc., Manson, WA, USA) in the MATLAB Version 7.3 (2013) (The Mathworks Inc., Natick, MA, USA) environment.

## 3. Results

We enrolled 196 patients, referred to the University Campus Bio-Medico of Rome between 2017 and 2019, who were affected by an ovarian mass and suitable for pelvic surgery. According to inclusion and exclusion criteria, the following 87 patients were not eligible for our study: 15 who were older than 80 years old, 3 who did not have a good performance status (ECOG < 2), 22 who did not sign the written informed consent, 21 who were younger than 18 years old, and 26 who were excluded because of an inadequate blood sample, renal or liver dysfunction, synchronous cancer, pneumonia and anesthesiology contraindication to surgery. Among the remaining 109 patients, 16 patients were excluded due to technical problems related to each single measurement. Therefore, the first analysis was performed on a total of 93 patients, specifically, 43 patients belonging to the control group and 50 to the tumor group. In a second step, to increase sensitivity and specificity, a sub-analysis was performed excluding, from the control group (43 pts), a total of 14 patients with fibroids and, from the group of tumors (50 pts), a total of 19 patients with tumor recurrence.

As a consequence, only 60 suitable women were taken into consideration for this sub-analysis, for a total of 29 control patients and 31 patients with cancer. The characteristics of patients and masses are listed in Table 1, Table 2, Table 3, Table 4 and Table 5. Table 1 shows that the mean age between cases and controls was 60 years old, most of the patients were in menopause, the average size of the mass was 7 cm, the mean BMI was 24, a small number of women smoked, and all the cases had altered CA125 and HE4. Table 2, Table 3 and Table 4 regard the cases and indicate that most of the women had a serous histotype (72%) stage III naïve, G3. Table 5 regards the controls and indicates that the most recurrent histotypes were fibroids, endometriomas, and serous cysts.

The analysis was divided into three phases as below:

In the first phase, a supervised approach was followed considering the following two groups: controls vs. k-ovarian diseases. In this case, the data fusion obtained from different sensors was considered, and the data were processed by a partial least square (PLS).

**Figure 4:**
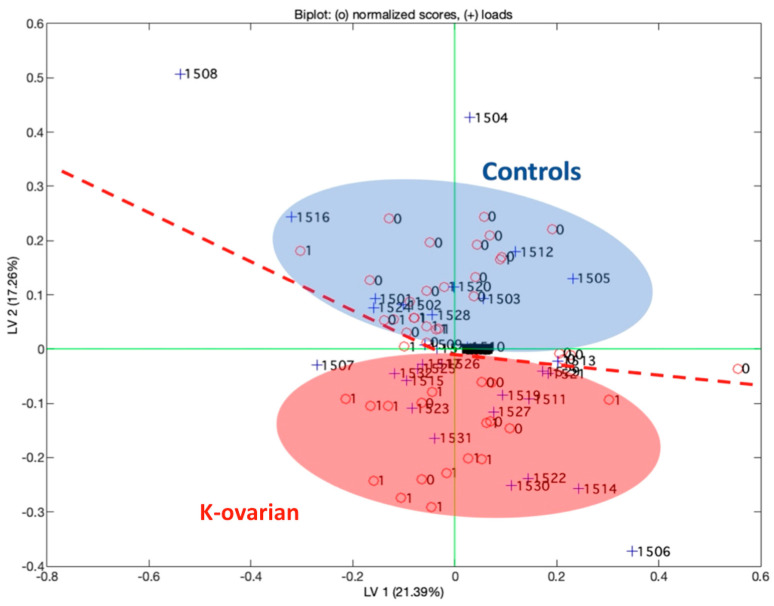
First two latent variables of the partial least square model. The biplot reports the loadings for the sensors used in the analysis.

Figure 4 represents the scores plot of the first two latent variables of the partial least square discriminant analysis model (PLS-DA). In the scores plot, the label “0” represents control subjects (Controls), while the label “1” represents subjects affected by neoplastic disease (K). In the figure, Latent Variable 2 (LV2) distinguishes the two groups, and the capability to classify the two groups is reported in the confusion matrix (Table 6) obtained with the same model. So, from Figure 4, it is possible to determine the ability of the multisensorial system to discriminate the two classes. These results evidence that there is not a single sensor that is able to specifically characterize one or the other class. The power of this approach is in using more sensors to discriminate a pathology. The number of sensors used in this analysis is 1532. The PLS-DA model was obtained using a data fusion among different kinds of sensors including 500 values related to electrochemical sensors for blood, 500 related to urine, 500 related to plasma, and 32 related to the responses of the gas sensors (QMB).

In the second phase, different predictive models were built. In the first model, 93 measurements were used, referring to 43 controls and 50 k-ovarian diseases. In this case, a supervised approach based on partial least square discriminant analysis was used. The leave-one-out criterion was performed for the validation model.

In Table 6, a confusion matrix is reported.

**Figure 5:**
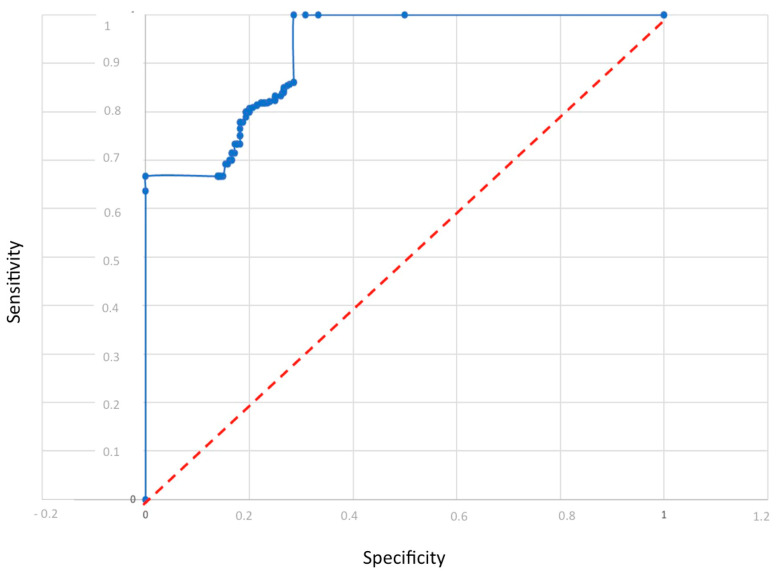
Model performance by ROC curves.

From the model, the sensitivity and specificity were calculated as follows: sensitivity: 86% and specificity: 88%. These values show the ability of the multisensorial device to discriminate between a healthy and an ovarian cancer-affected patient. The analysis demonstrates that this type of sensor can support a clinical decision (Figure 5 shows the model performance by ROC curves). Finally, an analysis without patients with tumor recurrence and fibroids was performed. In this case, a total of 60 measurements were considered, specifically 31 control subjects and 29 K-ovarian subjects (Table 7).

It is important to highlight that the single models obtained considering just a single biological fluid, among the fluids analyzed, are not significant in terms of the percentage of correct classifications. This means that each body fluid is not able to discriminate the presence of the pathology. Indeed, it is important to underline that the novelty and the potential of the method proposed here consist of the significance of all the data collected by each individual, which represents a multisensory and multi-specimen pattern. This is because of the fact that each sensor is not selective to specific compounds (solution or VOCs). Thus, each biological fluid is not able to provide specific information for the pathology if analyzed with a single sensor. Table 8 and Table 9 report the matrix for each kind of sensor. It is important again to note that the percentage of correct classification for each device is lower than the percentage obtained with the overall data.

## 4. Discussion

Our study demonstrates that our system can be used to better detect ovarian cancer, along with traditional methods such as clinical examination, serum biomarkers (Carbohydrate Antigen 125 (CA 125) and Human Epididymis Protein 4 (HE4)), transvaginal ultrasound (TVU), computed tomography scan (CT), and positron emission tomography (PET). Indeed, all these traditional methods have several limitations [4,31], and there is a concrete need to find an effective screening test for ovarian cancer to obtain a strong and real prevention. Many scientists have recently focused their attention on volatile organic compounds (VOCs) based on the original studies in which Horvath et al. demonstrated that human ovarian cancer has a specific odor that can be detected by a trained dog [16,17,18]. Since dogs are not suitable for clinical practice, Horvath used an electronic nose to accomplish the same task, with a sensitivity of 84.4% and a specificity of 86.8% in tissue tests. The e-nose idea was further discussed and investigated by other scientists, such as Raspagliesi et al. [26]. His in vivo study divided patients into three groups including ovarian cancer cases, benign masses, and controls, and the results reached a sensitivity of 89% and specificity of 86% when the ovarian cancer cases were compared with the benign masses and controls. However, better data were obtained when comparing ovarian cancer cases to controls, with a sensitivity of 98% and specificity of 95%. Another study by Haick et al. tested a nanoarray [24,25] to examine exhaled breath samples from ovarian cancer patients [24,25]. The nanoarray measured volatile organic compounds with good sensitivity (79%) and a 100% specificity in detecting ovarian cancer patients vs. controls. This shows that, in the case of a positive result, there is a high probability of ovarian cancer. In the case of a negative result, further tests can be avoided. It is essential to remember that the gold standard for the analysis of VOCs in exhaled breath is gas chromatography and mass spectrometry, which allow for both quantitative and qualitative analysis [32,33]. However, expensive equipment, laboratory setup, trained personnel, and the time-consuming process are the main limitations of this method [32,34]; this is why we opted for sensor array analysis, which is also the technique described in the research we mentioned before. Sensor array analysis and the e-nose are easy to use, are small-sized devices, and there is a direct acquisition of results and immediate analysis. As a limitation, they can only identify a fingerprint by multivariate analysis, while mass spectrometry can identify unknown VOC metabolites from samples [33,35]. Over time, not just the electronic nose but also the electronic tongue has started to be used in the detection of bladder and prostate cancer with promising results [29,30]. The electronic tongue used by Pascual in prostate cancer patients distinguished cases from controls with a sensitivity of 91% and a specificity of 73% [29], which is even higher than the prostate-specific antigen (PSA) test in blood. Encouraged by all this supportive literature, we decided to perform a similar study at our university, in which we used specific gas and liquid sensor arrays to discriminate ovarian cancer from benign masses. In fact, for the first time in the literature, our study merged the e-nose data from breath samples with the e-tongue data from urine, blood, and plasma samples in ovarian cancer cases versus controls. 

In addition, we compared our results to those obtained by the blood dosage of CA125 and HE4, which are serum markers currently used to identify ovarian cancer. These two, taken together inside the ROMA score, reach a specificity of 76,4% and a sensitivity of 95% [31]. Unfortunately, in our preliminary analysis, we found that these biomarkers do not provide a specific fingerprint to be read by sensors. Therefore, they should be used in association with the sensor array analysis to increase specificity. This means that if the CA125 and HE4 values are high, the probability of our system having identified a cancer is reinforced. In the future, it could be useful to investigate this correlation in a larger sample to better understand whether serum tumor biomarkers and sensors array can become part of a diagnostic algorithm for the non-invasive screening of ovarian cancer patients.

We compared our results with those obtained by ultrasounds and found a better sensitivity (89% vs. 88%, respectively) and specificity (87% vs. 84%, respectively). On the other hand, comparing our results to other imaging techniques (computed tomography scan (CT) and positron emission tomography (PET)), we obtained a much better sensitivity (89% vs. 78% CT/PET) and specificity (87% vs. 68% CT/PET) [7,31].

According to the data, our analysis can reach a similar sensitivity and specificity to serum biomarkers, ROMA SCORE, and ultrasounds and a higher sensitivity and specificity in comparison with CT and PET. Raspagliesi et al. also stated that the e-nose can achieve the same or even higher specificity values than CA125 and ultrasounds [26]. Unfortunately, it is clear that no method could reproduce the sensitivity (100%) and specificity (97.5%) of a trained dog [16,17,18], and further research is needed to scientifically reproduce such capability. 

## 5. Conclusions

The present study has as its most relevant strength the potential to work alongside the currently available diagnostic techniques (ultrasounds, blood biomarkers, and PET CT) to determine the nature of ovarian masses and enhance their sensitivity and specificity. All this can be used in the context of screening, diagnosis, and follow-up of ovarian cancer.

In parallel, this study has some limitations. For example, all the VOCs were collected from patients inside the hospital, but the influence of environmental air was not monitored. This might constitute a potential bias and could be a further refinement in our next study. Another limitation is that an independent cohort was not used for model validation, and this could unfortunately affect the predictive performance of the system. This has to be improved in our further research. In addition, the impact of individual comorbidities on misclassification exploited by sensor arrays on the discrimination of ovarian cancer from benign masses was not investigated and therefore quantified.

It would be advisable to deepen this aspect to better understand which characteristics belonging to the patients (chronic metabolic, autoimmune, or infectious diseases) can make the method more or less effective. At the same time, it could be useful to investigate whether some aspects belonging to the neoplasm could also contribute to the classification that facilitates its more correct identification. 

Another interesting aspect is to examine the potential capability of the tool to differentiate ovarian cancer histotypes or the FIGO staging. This aspect was not taken into consideration in our study, while the research by Raspagliesi et al [26] suggested possible e-nose discrimination between early-stage and advanced-stage ovarian cancers and controls and also between the various histologies. 

In conclusion, this is the first time in the literature that the efficacy of sensor array analysis in blood, urine, and breath samples together has been analyzed. Our preliminary results suggested the potential role of sensor array analysis for the detection of OC in a selected group of patients. Starting from the present study, in the future, various investigation scenarios could open up both in the context of screening and follow-up of ovarian cancer.

For screening, the next step may consist of the investigation of altered VOCs in a larger-scale population sample to determine whether they could be predictive of a higher risk of developing ovarian cancer in comparison to non-pathological VOCs. This may allow for the identification of patients at higher risk, regardless of the current presence of ovarian masses. 

Another interesting field of application could be the follow-up of patients who underwent surgery or medical therapy for ovarian malignancies to verify the eventual post-surgery modifications in neoplasm metabolic and biochemical patterns and any supervening changes that are indicative of relapse or progression of the disease.

In addition, VOCs could be used to monitor disease response during treatment, thus discriminating between responders and non-responders. This has already been tested in lung cancer patients during immunotherapy by De Vries et al. [36] and should be proposed in ovarian cancer during chemotherapy.

## Figures and Tables

**Table 1:** Patients’ characteristics.

	TUMORS (50)	CONTROLS (43)	*p*-VALUE
AGE	60 (24–80)	59 (23–76)	0.22
MAIN SIZE of the mass (cm)	7 (3–10)	6.5 (2.8–10.5)	0.3
BMI, (mean)	24	24.9	0.49
MENOPAUSE (%)	27	22	0.01
CA125 (mean)	313 (5–2130)	28.3 (3–179)	<0.001
HE4 (mean)	906 (33–7287)	92 (37–144)	<0.001
SMOKE (N)	15	26	0.44
SURGERY (N)	100	100	0.1

**Table 2:** Histotypes of the tumors.

HISTOTYPE	N (%)
Endometrioid	5 (10%)
Serous	36 (72%)
Clear cell	1 (2%)
Germinal line or sexual cord	4 (8%)
Other	4 (8%)

**Table 3:** FIGO staging.

STAGE	N (%)
I	14 (28%)
II	9 (18%)
III	27 (54%)
Recurrence	19 (38%)
Naïve	31 (62%)

**Table 4:** Grading.

GRADING	N (%)
G1	5 (10%)
G2	4 (8%)
G3	41 (82%)

**Table 5:** Histotypes of benign masses.

HISTOTYPE	N (%)
Fibroid	14 (32%)
Endometrioma	10 (23%)
Mucinous	2 (4.6%)
Cystadenofibroid	3 (7%)
Endometrioid/adenofibroid	1 (2.3%)
Serous cyst	8 (18.6%)
Mature cystic teratoma	2 (4.6%)
Paratubaric cyst	3 (7%)

**Table 6:** Confusion matrix obtained by the PLS-DA model.

			Predicted
		Controls	K-ovarian
**Real**	Controls	37	6
K-ovarian		6	44

**Table 7:** Confusion matrix obtained by the PLS-DA model without relapsed subjects.

			Predicted
		Controls	K-ovarian
**Real**	Controls	26	3
K-ovarian		4	27

**Table 8:** Confusion matrix E-NOSE.

			Predicted
		Controls	K-ovarian
**Real**	Controls	33	10
K-ovarian		9	41

**E-NOSE:** sensitivity = 0.417 = 41%; specificity = 0.912 = 91%; percentage of correct classification = 79.57%.

**Table 9:** Confusion matrix E-TONGUE. Reproduced with permission from R. Angioli, et al.

			Predicted
		Controls	K-ovarian
**Real**	Controls	23	20
K-ovarian		11	39

**E-TONGUE:** sensitivity = 39/50 = 78%; specificity = 23/43 = 53%; percentage of correct classification = 66.67%.

## Data Availability

The original contributions presented in this study are included in this article. Further inquiries can be directed to the corresponding author.
